# Alternative Methods for Skin-Sensitization Assessment

**DOI:** 10.3390/toxics10120740

**Published:** 2022-11-29

**Authors:** Dominika Gądarowska, Joanna Kalka, Anna Daniel-Wójcik, Inga Mrzyk

**Affiliations:** 1 The Faculty of Energy and Environmental Engineering, Silesian University of Technology, Konarskiego 18, 44-100 Gliwice, Poland; 2 Łukasiewicz Research Network—Institute of Industrial Organic Chemistry Branch Pszczyna, Doświadczalna 27, 43-200 Pszczyna, Poland

**Keywords:** allergic contact dermatitis, risk assessment, skin sensitization, alternative methods

## Abstract

Skin sensitization is a term used to refer to the regulatory hazard known as allergic contact dermatitis (ACD) in humans or contact hypersensitivity in rodents, an important health endpoint considered in chemical hazard and risk assessments. Information on skin sensitization potential is required in various regulatory frameworks, such as the Directive of the European Parliament and the Council on Registration, Evaluation and Authorization of Chemicals (REACH). The identification of skin-sensitizing chemicals previously required the use of animal testing, which is now being replaced by alternative methods. Alternative methods in the field of skin sensitization are based on the measurement or prediction of key events (KE), i.e., (i) the molecular triggering event, i.e., the covalent binding of electrophilic substances to nucleophilic centers in skin proteins; (ii) the activation of keratinocytes; (iii) the activation of dendritic cells; (iv) the proliferation of T cells. This review article focuses on the current state of knowledge regarding the methods corresponding to each of the key events in skin sensitization and considers the latest trends in the development and modification of these methods.

## 1. Introduction

Allergic contact dermatitis (ACD) is an inflammatory dermatosis resulting from the repeated contact of many small-molecule chemicals which have the ability to activate the immune system in delayed-type hypersensitivity. A total of 15–20% of the general population suffer from ACD caused by one or more chemicals, most commonly nickel, fragrances and preservatives; however, pollution present in the environment can also contribute to the development of ACD [1,2].

Information on skin-sensitization potential is required by several regulatory regimes, most notably Registration, Evaluation and Authorization of Chemicals (REACH). This Regulation of the European Parliament and the Council aims to ensure a high level of protection for human health and the environment [3]. REACH also promotes alternative methods for risk assessment of chemicals that have become essential for modern toxicology and ecotoxicology.

**Figure 1:**
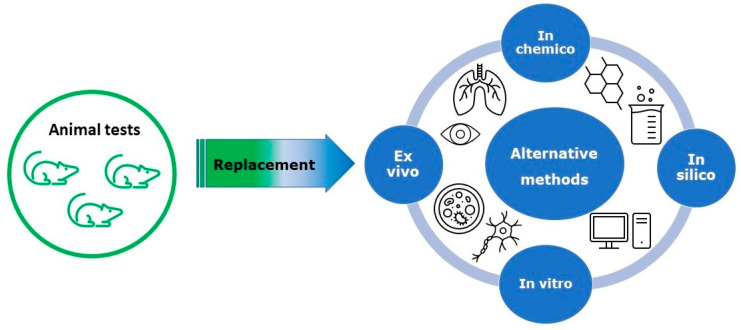
Basic systems used as partial or full replacement of animals.

There are four basic systems used as partial or complete substitutes for animals in toxicological experiments (Figure 1):

(i) In vitro methods, which are widely used in toxicology to assess topical toxicity, e.g., skin irritation/corrosion/sensitization, as well as systemic toxicity, e.g., genotoxicity and developmental and reproductive toxicology [4]. In these methods, the animal organism is replaced by a biological system consisting of cells or tissue models such as primary cultures, finite cell lines, continuous cell lines or reconstructed 3D tissues. In vitro systems are based on cell monolayer, coculture systems and three-dimensional (3D) tissues, taking into account the tissue microenvironment. There has been a lot of interest in recent years in the organs on chips (OoCs) which reflect the structure and physiological properties of tissues or organs.
(ii) Ex vivo methods (isolated animal tissues and organs). In toxicology, ex vivo methods are mainly used to assess corrosion. Eye corrosion is evaluated by the methods adopted by the Organization for Economic Cooperation and Development (OECD) that use eyes obtained from slaughtered chickens (OECD TG 438) or cattle (OECD TG 437) [5,6]. In turn, skin corrosion is assessed by a rat skin transcutaneous electrical resistance (TER) test (OECD TG 430). This method uses skin fragments obtained from humanely euthanized rats [7]. Precision-cut tissue slices (PCS) are also used, both from rodents and from humans, e.g., from biopsy material [8].
(iii) In chemico methods (chemical methods in which the properties of the substance under test are evaluated by reaction with appropriate material, without using human or animal cells). In chemico methods are used to evaluate reactive substances. In order to develop them, it is necessary to know about the interaction of the test substance with a biological substance. These methods are widely used in the skin-sensitization area.
(iv) In silico methods: computer simulations and mathematical models such as Quantitative Structure–Activity Relationship (QSAR), Threshold of Toxicological Concern (TTC), etc. [9]. In silico methods are used to predict toxicity based on computational methods and are used in conjunction with other alternative methods to reduce in vivo testing. The particular advantage of in silico methods is the possibility of assessing the potential physico-chemical or biological properties of substances before they are synthesized [10,11,12].

The replacement of animal tests has been strongly advocated [13], especially when validation research has provided evidence that the new approaches do not lower safety standards and can be integrated into regulatory safety assessments [14].

Apart from ethical issues, the use of animal models in toxicological research, including skin sensitization, is also associated with the problem of reproducibility and sensitivity of the model used for the study. As reported by Maertens et al. [15], the repeatability of the test for maximization in guinea pigs (GPMT) obtained for 624 samples in the test was 93%, while the repeatability of the Local Lymph Node Assay (LLNA) in mice (for 296 samples) was 89%. It should also be kept in mind that there is no ideal animal model, and there are also some differences in sensitivity and predictive ability between the animal species used as test models. For example, the agreement of the responses and thus the classification of the results in the test with mice and guinea pigs was 77% in 403 samples. Thus, this is another reason to search for alternative methods to better predict the effects of chemical compounds on humans and the environment.

In this article, we present the current knowledge on available alternative methods corresponding to each of the key events in skin sensitization. Additionally, coculture methods are briefly discussed. Because both the system of good laboratory practice and the integrated approach to testing and evaluation is constantly being improved, every effort has been made to include only the most recent, corrected and updated versions of procedures and methodologies in the article.

## 2. Skin-Sensitization Mechanism and Standard Assessment

Contact allergens (haptens) are small, reactive molecules with molecular weight below 500 Da. They are not immunogenic by themselves; the immune system recognizes them after binding to peptides and proteins [16,17]. Recent literature data show that the size of haptens is not a crucial parameter for skin sensitization, but their reactivity [18], which is related to unpaired electrons in the last layer of the molecule. Haptens are electron acceptors (electrophiles) that react by covalent bonding with electron-rich amino acids (nucleophiles) such as lysine, cysteine, histidine, methionine or tyrosine [19,20]. Some chemicals with low molecular weight become sensitizers after abiotic activation (prehaptens) or metabolic transformation (prohaptens) [21,22]. Abiotic transformation occurs outside the skin and involves the conversion of haptens without the involvement of a specific enzyme system, for example, by oxidation or photoactivation [23]. Metabolic activation occurs in skin containing appropriate enzymes. Changing the chemical structure of xenobiotics increases hydrophilicity and allows excretion from the body.

**Figure 2:**
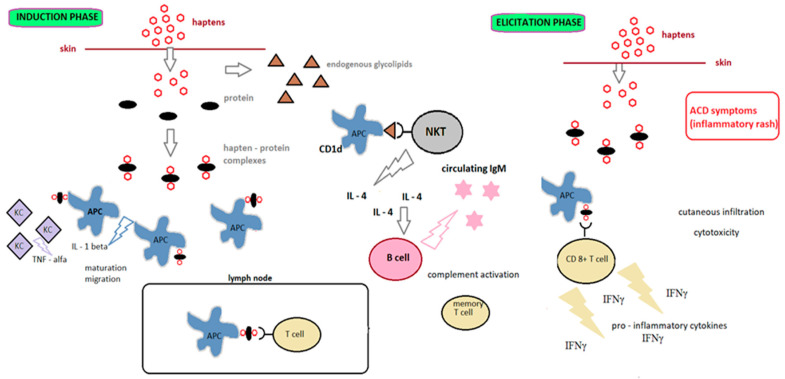
Mechanism of allergic contact dermatitis [24]. KC: keratinocytes, APC: antigen-presenting cells, NKT: natural killer T, IFNγ: interferon γ.

ACD occurs in two phases: induction (also known as afferent) and elicitation (also known as efferent). As shown in Figure 2, the induction phase encompasses all phases, from the initial contact with the allergen to the development of sensitization. The elicitation phase begins after contact with the hapten of an individual who has already been sensitive and leads to ACD [20].

Since allergic contact dermatitis is considered to be the most prevalent form of immunotoxicity found in humans [25], it is important to evaluate the sensitizing potential of chemicals. The United Nations Globally Harmonized System of Classification and Labelling of Chemicals (UN GHS) distinguishes substances into category 1 (sensitizing substances) and no category (nonsensitizing substances, not classified). Substances are classified as skin sensitizing if there is evidence that the substance may cause sensitization by skin contact in a significant number of humans or if positive results have been obtained in a corresponding animal test (UN GHS). Within the OECD, there are four main guidelines for standardized skin-sensitization tests:

- OECD 406: Skin Sensitization Guinea Pig Maximization Test and Buehler Test [26]
- OECD 429: Skin Sensitization—Local Lymph Node Assay (LLNA) [27]
- OECD 442A: Skin Sensitization—Local Lymph Node Assay: DA (LLNA:DA) [28]
- OECD 442B: Skin Sensitization—Local Lymph Node Assay: BrdU-ELISA or FCM [29]

The OECD 406 Guideline is intended for use with guinea pigs, while the rest of the guidelines are intended for use with mice. The LLNA in vivo assay has largely replaced the guinea pig-based tests [30] and offers many advantages, especially the reduction and refinement of animal use [31]. It is based on the induction of primary lymphocyte proliferation in the auricular lymph nodes draining the area of the tested substance application. Lymphocytes’ proliferation is proportional to the applied dose and enables the measurement of sensitization by radioactive labeling. The other two methods are nonradioactive modifications of LLNA assay that measure lymphocytes’ activation by bioluminescence (LLNA:DA) or ELISA (BrdU-ELISA) and flow cytometry (BrdU-FCM) methods.

Where sufficient data are available, a subclassification of category 1 substances should be made according to potency. Subcategory 1A—substances that are very common in humans and/or have high potency in animals and can be expected to cause significant sensitization reaction in humans. Subcategory 1B—substances with low or moderate frequency of occurrence in humans and/or low or moderate potency in animals and which may be expected to cause sensitization reaction in humans. In general, subcategorizations are based on data from animals or humans [3].

## 3. Skin-Sensitization Assessment—Alternative Methods

Currently, the trend is to reduce the number of animal tests or to replace these tests with alternative methods. According to the regulation REACH, in vivo tests are performed only when in vitro/in chemico testing methods are not applicable, or the results of these tests are not suitable for classification and risk assessment [32]. Alternative methods are based on existing knowledge describing the effects of molecular perturbations at the subcellular, cellular, tissue, organ, whole animal and population levels and integrated in Adverse Outcome Pathways (AOP). In the AOP of cutaneous sensitization, the following key events (KE) can be distinguished:

- KE1: the first key event, i.e., the molecular initiating event (MIE), is the covalent binding of electrophilic substances to nucleophilic centres in skin proteins;
- KE2: the second key event, i.e., the keratinocytes (KCs) activation;
- KE3: the third key event is the activation of dendritic cells (DCs);
- KE4: the fourth key event is Tcell proliferation.

At the moment, it is not possible to replace the evaluation of sensitization with animals using one alternative method addressing the first three key events to provide chemical safety assessment.

For many years, there has been consensus in the research community that the best approach to assessing skin-sensitization potential is based on multiple sources of information that integrate, weigh all relevant existing evidence and guide the targeted generation of new data as needed [4]. In addition, most single tests have some specific limitations that can be overcome when combined into a research strategy. These limitations include:

- Unsatisfactory accuracy and predictability;
- Not all possible outcomes of interest are included in a single test;
- The need to consider different modes of action;
- In vitro tests represent only one or a few steps in complex in vivo processes;
- Not all classes of test substances are covered;
- Not all classes of severity of effects are covered;
- Positive test results are rare, and the number of false positives is too high;
- Lack of integrated data and evidence from different studies;
- Lack of integrated information on absorption, distribution, metabolism and excretion of test substances, which makes it impossible to extrapolate data from in vivo studies [4].

To overcome those limitations, data from different sources of information (in chemico, in vitro, in silico methods; read-across predictions from chemical analogues; existing human data such as epidemiological data, Human Repeat Insult Patch Test (HRIPT), clinical data; existing animal test data) are used within the integrated approaches to testing and assessment (IATA) or defined approaches (DAs) to obtain information on whether the test substance is a skin sensitizer and what the skin sensitization potency is [33].

The assessment of sensitization potential within IATA is based on weighted multiple information and expert judgment under weight of evidence (WoE) and is always requested for regulatory decisions [4]. An integrated analysis of existing information coupled with the generation of new information using testing strategies is flexible and allows adaptation to regional legal regulations and requirements [33]. In turn, DAs generate a prediction without the expert judgement using the fixeddata interpretation procedure (DIP), i.e., mathematical models or rules-based approaches [34,35]. In 2021, the OECD guideline No. 497 was adopted, which describes a defined approach allowing for a prediction of hazard identification and/or hazard characterization. As indicated in Table 1, three DAs are included in the guideline. Furthermore, 2o3 DA provides final prediction based on two concordant results from study, covers at least two of the first three KE and allows for hazard identification, i.e., discrimination between skin sensitizers and nonsensitizers. Integration of computational methods with experimental methods increases the prognostic accuracy [36]. Only the combination of in chemico/in vitro sources of information with in silico methods (DEREK Nexus v6.1.0; OECD QSAR Toolbox v4.5) allows for hazard as well as potency identification (ITSv1 DA; ITSv2 DA).

DA limitations are related to the limitations of individual in chemico/in vitro/in silico methods that have been evaluated using monoconstituent substances rather than mixtures or formulations [37]. Much more work is needed for evaluating complex mixtures and formulations; therefore, the next-generation risk assessment approaches for skin sensitization are needed [35]. As more in vitro assays are developed and evaluated, there will be opportunities to include these data in the new approach [38].

A key issue in assessing the predictive capacity of alternative methods and defined approaches is the selection of appropriate reference data [39]. For skin sensitization, gold standard, LLNA data are most often used. However, an analysis of 12 different DA/IATAs showed that all of them allow hazard and potency identification equal to or better than the LLNA. The authors pointed out that comparing DA predictions with animal prediction may underestimate the accuracy of DA models that are in many cases more efficient at predicting skin sensitization in humans [40]. Natch et al. emphasize that the optimal approach to assess the predictive capacity of alternative methods and defined approaches is to consider both animal and human data, especially since in the case of skin sensitization, unlike other areas of toxicological endpoints, human data are accessible [41]. There is a need for continuous evaluation of DA/IATA versus animal and human data to optimize the use of nonanimal data for skin-sensitization risk assessment [42].

It should be noted that the regulatory requirements for the assessment of skin sensitization may differ depending on the country and the type of tested substance. Although many regulators accept alternative methods, only some of them indicate specific methods. Yet knowing the demands of regulatory bodies is essential in order to further refine methods and implement new methods and integrated approaches. The skin-sensitization requirements under REACH are best described [43]. In the following subsections, practical implications of key event-based methods are described.

### 3.1. Key Event 1 (KE1)-Based Methods

The internationally recognized guideline addressing KE1, i.e., molecular initiating event (TG 442C), has been adopted by the OECD and includes three in chemico methods: Direct Peptide Reactivity Assay (DPRA), Amino acid Derivative Reactivity Assay (ADRA) and The kinetic Direct Peptide Reactivity Assay (kDPRA) [44].

DPRA uses synthetic peptides containing lysine and cysteine, which are incubated for 24 h with an excess of chemicals. Subsequently, the concentration of peptides is evaluated by high-performance liquid chromatography with UV-detection (HPLC-UV). Substances are classified into one of four classes of reactivity on the basis of the cysteine and lysine peptide depletion value. Since DPRA uses peptides with low UV absorption [45], the final concentration of the tested chemical is high (100 mM), which prevents the analysis of poorly soluble substances. Additionally, the peptides are detected at 220 nm, similarly to various substances, which can lead to coelution and imprecise measurement of peptides [46]. These disadvantages find a solution in the ADRA method, in which two types of detection are used: ultraviolet (UV) detection and fluorescence (FL) detection. Using two amino acid derivatives, N-(2-(1-naphthyl)acetyl)-L-cysteine (NAC) and α-N-(2-(1-naphthyl)acetyl)-L-lysine (NAL) [45,47], which are highly sensitive nucleophilic reagents, allowed us to reduce the final concentration of the tested chemical [48] and study hydrophobic substances [45]. For a substance with a known molecular weight (monoconstituent substance, mixture, multiconstituent substance of known composition), ADRA should be performed using a stock solution at a concentration of 4 mM. However, a gravimetric approach based on a stock solution prepared at 0.5 mg/mL should be performed for a monoconstituent substance or a mixture of unknown molecular weight [44].

Recent studies confirmed the possibility of using ADRA to evaluate mixtures used in cosmetic ingredients, thanks to the application of the weight concentration, not the molar one [49]. Since UV detection at 281 nm is used, even if the tested chemical is coeluted with the nucleophilic agent, there will be no apparent effect on the quantification of the peptides as long as the tested chemical itself does not absorb UV at 281 nm. Only test chemicals containing conjugated double bonds of a certain length absorb UV at these wavelengths [50]. However, NAC is susceptible to oxidation, especially in the presence of even a small amount of metal ions. It can be prevented by adding a low concentration of ethylenediaminetetraacetic acid (EDTA) [51]. The analysis of multiconstituent substances may also be possible with fluorescence detection (ADRA-FL method), as the number of peaks in the chromatogram has been significantly reduced. Fujita et al. reported that fluorescence detection for HPLC analysis of NAC and NAL reveals a single sharp peak and a stable baseline [52].

DPRA and ADRA support discrimination between skin sensitizers and nonsensitizers. As a recently introduced method, kDPRA allows discrimination of subcategory 1A skin sensitizers from those not categorized as subcategory 1A (non-subcategory 1A), i.e., subcategory 1B or no category, but does not allow the distinguishing of sensitizers (Category 1) from nonsensitizers [44]. The kDPRA uses five substance concentrations (5, 2.5, 1.25, 0.625 and 0.3125 mM) at six reaction times (10, 30, 90, 150, 210 and 1440 min) and multiwall plate fluorometric read-out only for the Cys-peptide.

There have been a variety of detection methods as well as types of nucleophiles used (Table 2). Cor1C420 assay based on both liquid chromatography-mass spectrometry (LC-MS) analysis and detection of free thiol groups allows simultaneous determination of peptide depletion, peptide oxidation (dimerization), adduct formation and thiol reactivity and thus generates a more detailed characterization of the reactivity of a molecule. A more-reactive peptide, containing both Cys and Lys electrophilic residues, is used, which allows a reduction of the concentration of both the test chemical and the test peptide, thus reducing solubility issues of test chemicals [46].

N-butylamine and 1-butanethiol as surrogates for nucleophilic amine and thiol groups of lysine and cysteine, respectively, were proposed in the NMR spectroscopy method. This method provides a rapid initial assessment of the reactivity of a chemical and allows the use of aqueous as well nonaqueous solutions. NMR spectroscopy can provide additional data about chemical reactivity information used to inform toxicological risk assessments [53]. In other NMR-based methods, a model thiol dansyl cysteamine (NMR-DCYA) was applied due to its distinctive NMR spectrum and minimal interference with the majority of electrophiles. A kinetics version of this method could be extended to obtain a reliable reactivity index for potency estimation, and the accessibility of such resulting rate constants may be useful for the quantitative prediction of potency by QMM or read-across methods [54]. DCYA was also used in a fluorescence-based method—high-throughput screening method (HTS-DCYA)—where the reaction adduct is quantified directly [55,56]. This method evaluates reactivity of the overall mixture even when the contributions of individual components is unknown (the fluorescence emission can be extrapolated as the total number of DCYA-adducts in solution). It was estimated that the HTS-DCYA method would be sensitive enough to enable the detection of highly reactive compounds present in a total concentration of 2–3% *w*/*w* [57]. The HTS-DCYA method may be useful in prescreenings of large chemical libraries before performing more costly and time-consuming in vitro and clinical evaluations of skin-sensitization potential [55]. In turn, high-performance liquid chromatography coupled with tandem mass spectrometry (HPLC/MS-MS-based DPRA) correctly predicted chemicals as sensitizers or nonsensitizers as well as increased the possibility of identifying substances noncovalently binding to peptides or chemicals with overlapping peaks with either peptide. The HPLC/MS-MS method deals better with weakly water-soluble compounds [58].

In contrast to methods requiring expensive equipment, spectro-DPRA assay uses the spectrophotometric method to monitor peptide reactivity. Unreacted cysteine-containing peptide and the amine group of unreacted lysine-containing peptide are detected by UV-VIS spectrophotometer and fluorometer, respectively. The spectrophotometric values were measured before and after the addition of detection reagents to avoid any interference from background signals. The main advantage is the reduction of the measurement time to 10 min compared to DPRA (22–26 h); however, this method could not estimate pre/prohapten and was unable to predict highly lipophilic chemicals [59,60,61]. The problem with the correct prediction of prohaptens also appears in the method using glutathione and cysteamine instead of synthetic peptides [62]. Cysteamine and glutathione residues following the incubation with chemicals were derivatized with 4-(4-dimethylaminophenylazo) benzenesulfonyl chloride (DABS-Cl) and formed very stable (up to 48 h) complexes. Since sodium acetate buffer at pH 4.5 is used, there is a risk of incorrect results for highly basic chemicals. This method can be applied to differentiate skin sensitizers from nonsensitizers. To increase the potential of prohaptens detection, a horseradish peroxidase and hydrogen peroxide (HRP/P) oxidation system was included in the peroxidase peptide reactivity assay (PPRA) method. This work shows the potential to incorporate an enzyme-mediated activation step for prohaptens into an in chemico skin-sensitization assay that results in the detection of all types of sensitizers [63].

PPRA has also been checked regarding whether it can detect respiratory allergens; however, it does not show an advantage over the DPRA method in distinguishing between respiratory and skin sensitizers [64].

An example of a method which fulfils the element of pathway associated with protein expression is the allergen–peptide/protein interaction assay (APIA). APIA focuses on the initial haptenation processes in human skin and evaluates interactions between the potential allergens and skin-related proteins or peptides [65]. APIA is carried out under conditions imitating the distinct human epidermal reactivity compartments of the skin surface (pH 5.5), stratum basale (pH 6.8) and typical physiological conditions (pH 7.4) [66].

Electrophilic allergen screening assay (EASA) uses two complementary probes: an amine-based probe, pyridoxylamine (PDA), and a thiol-based probe, 4-nitrobenzenethiol (NBT), which represent lysine and cysteine side chains [67,68]. Initially, the method was developed using a cuvette format. Then, a 96-well plate format was used to increase throughput and include control measurements. The inclusion of multiple control measures contributed to the identification and correction of systematic errors. Absorbance for NBT and absorbance as well as fluorescence for PDA was recorded at approximately 5 min ± 30 s, 20 ± 2 min, 35 ± 2 min and 50 ± 2 min. Using 92 testing chemicals, the researchers achieved compliance of the EASA results with LLNA and GPMT comparable to DPRA, i.e., 73% [69].

The in vitro test using the THP-1 cell line, which is widely used in the KE3 methods, was also used to assess KE1. The SH-test is based on the measurement of cell-surface thiols changes after two hours of treatment with chemicals. A nonpermeable thiol-reactive compound, Alexa Fluor 488 C5 maleimide (AFM), is used to detect changes of cell-surface thiols induced by binding of haptens to cell-surface proteins [70]. Recently, the SH-test was improved by developing a new decision-making system, changing the statistical method and evaluating and determining the maximum number of repetitions necessary for optimal efficiency. In contrast to the DPRA, the SH test can be used for evaluating metal compounds and is easily applied to substances of unknown molecular weight and mixtures because it uses a gravimetric measurement method to set assay concentrations [71].

**Table 2:** KE1. Summary of methods.

Assay	Detection Method	Type of Nucleophile	Dataset	Accuracy [%]	Specificity [%]	Sensitivity [%]	Source /Ref.
				Compared to the LLNA			
DPRA	HPLC-UV	synthetic peptide containing cysteine and lysine	157	80	77	80	[44]
ADRA	HPLC-UV	NAC, NAL	136	76	79	76	[44]
kDPRA	Fluorescence	cysteine peptide	180	85	86	84	[44]
ADRA-FL	HPLC-FL	NAC, NAL	47	-	-	-	[52]
COR1-C420	LC-MS	COR1-C420	80	88.8 *	89.5 *	88.5 *	[46]
NMR	NMR spectroscopy	n-butylamine, 1-butanethiol	8	-	-	-	[53]
NMR-DCYA	NMR spectroscopy	DCYA	17	-	-	-	[54]
HTS-DCYA	Fluorescence detection	DCYA	33	82	90	78	[55]
HPLC/MS-MS method	HPLC/MS-MS	cysteine and lysine peptide	18	-	-	-	[58]
PPRA	HPLC/MS-MS	cysteine peptide	15	-	-	-	[63]
APIA	MALDI-TOF mass spectrometry	peptide-21 and peptide-20	3	-	-	-	[66]
Spectro-DPRA	UV-VIS spectrophotometry/ fluorometry	cysteine and lysine peptide	40	82.5	86.7	80	[60]
Method using small, endogenous molecules	HPLC—PDA	cysteamine, gluthatione	30	93	82	100	[62]
EASA	absorbance and fluorescence	4-nitrobenzenethiol (NTB) pyridoxylamine (PDA)	92	-	-	-	[69]
SH test	flow cytometry	cell surface thiols	52	-	-	-	[70]
Improved SH test	flow cytometry	cell surface thiols	25	84	62.5	94.1	[71]

* For depletion value.

### 3.2. Key Event 2 (KE2)-Based Methods

Keratinocytes activation can be evaluated by methods assessing the activation of biochemical pathways, analysis of gene and protein expression, and evaluation of proinflammatory cytokines. The secretion of inflammatory cytokines is recognized as being responsible for the pathological hyperactivation of cells, called excitotoxicity. Manifestation of excitotoxicity is most prominent in the cytokine storm syndrome [72].

Several methods are based on the measurement of Nrf2 pathway activation (Table 3). Activity of Nrf2 transcription factor, a major regulator of oxidative and electrophilic stress, is negatively regulated by Kelch-like ECH-associated protein 1 (Keap1) [73], which is the target for haptens [74]. Conformational changes in the Keap1 lead to accumulation of Nrf2, resulting in translocation into the nucleus and activation of antioxidant response element (ARE)-depending genes [75,76,77,78]. There are two validated assays included in the OECD guideline No. 442D, i.e., the ARE-Nrf2 luciferase KeratinoSens^TM^ and the ARE-Nrf2 luciferase LuSens. Positive results generated with these methods may be used on their own to classify a chemical into UN GHS category 1 [79]. Immortalized adherent cell lines derived from human keratinocytes (KCs) were used for both assays. The luciferase reporter gene was permanently introduced into KCs under control of the ARE-element of the human *AKR1C2* gene (ARE-Nrf2 luciferase KeratinoSens^TM^ assay) [76] or the rat *Nqo1* gene (ARE-Nrf2 luciferase LuSens assay) [80]. Quantitative evaluation of luminescence after induction of the luciferase gene in cells after exposure to test substance is an indicator of the activity of transcription factor Nrf2 [79]. Furthermore, cell viability is determined with 3-(4,5-dimethylthiazol-2-yl)-2,5-diphenyltetrazolium bromide (MTT). Resazurin-based viability assessment, not requiring cell lysis, was proposed as an alternative measurement or instead of the MTT test [81,82]. Usage of the KeratinoSens™ assay with agrochemical formulations demonstrated promising results [83]. All examined active ingredients (n = 8), three of four sensitizing formulations, and all six nonsensitizing formulations were correctly predicted comparing to in vivo data. However, it was necessary to modify the standard assay procedure whereby the formulation was assumed to have a common molecular weight. Other researchers showed the possibility of using KeratinoSens™ to assess the sensitizing effect of plant extracts [84]. Moreover, the KeratinoSens^TM^ and h-CLAT (KE3 method) binary test battery have greater sensitivity for detection of minute amounts of sensitizer than LLNA [85].

For detection of skin sensitizers in medical devices, a reporter cell line MDA-ARE, characterized by high levels of Nrf2 and Keap1, was created. It allowed reduction of incubation time and application of phosphate-buffered saline (PBS) as an induction medium without influence on cell viability and proliferation [86].

To increase the possibility of haptens and prohaptens detection, rat S9 liver fraction alone [87] or supplemented with human skin cytochrome P450 enzymes [88] found application in KeratinoSens^TM^. Since only a small fraction of the known skin sensitizers need to be activated, the KeratinoSens-S9 assay is proposed only for chemicals negative in a first screening or containing either phenolic groups and/or alkoxy groups attached to a benzene ring, aromatic amines and conjugated dienes [87]. In turn, to increase the sensitivity, ARE cell line derived from the human MCF7 breast carcinoma cell line containing an eightfold repeat of the ARE sequence upstream of luciferase gene was used. The AREc32 cell line assay is characterized by good sensitivity to identify moderate, strong and extreme allergens and high specificity [89].

**Table 3:** KE2. Biochemical pathway based methods.

Method	Cell Line	Dataset	Accuracy [%]	Specificity [%]	Sensitivity [%]	Source /Ref.
			Compared to the LLNA			
ARE-Nrf2 Luciferase KeratinoSens^TM^ Test	KeratinoSens™ transgenic cell line	145	77	72	79	[79]
The ARE-Nrf2 Luciferase LuSens test	LuSens transgenic cell line	72	74	74	74	[79]
AREc32 cell line assay	AREc32	102	83	86.6	81.4	[89]
MDA-ARE assay	MDA-ARE reporter cell line	22	-	100	92	[86]
Keratinosens^TM^-resazurin assay	KeratinoSens™ transgenic cell line	35	-	-	-	[81]
Keratinosens – S9 assay	KeratinoSens™ transgenic cell line	77	-	-	-	[87]
Keratinosens assay with S9 and P450 assay	KeratinoSens™ transgenic cell line	2	-	-	-	[88]

As shown in Table 4, an analysis of various genes’ expression has been used with 2D or 3D culture models. The 2D model, i.e., normal human keratinocytes (HaCaT), was used to analyze the genes involved in the following pathways: Keap1/Nrf2/ARE/EpRE, ARNT/AhR/XRE and Nrf1/MTF/MRE, by quantitative real time polymerase chain reaction (qRT-PCR). The main limitation is its inability to separate nonsensitizers from very weak sensitizers [90]. The same test system was used in the HaCaT gene profiling method [91]. After 4 h exposure of HaCaT cells, the expression of genes of oxidative stress response (*HMOX1*, *STC2*, *ADM* and *SRD1*) and genes related to the inflammatory response (*cFOS* and *FosL1*) was analyzed using real-time polymerase chain reaction (RT-PCR).

In the SenCeeTox method, HaCaT cells were replaced by the 3D model—the reconstructed human epidermis (RhE) [92]. The RhE mimics skin structure and organization, has a differentiated epidermis and horny layer (stratum corneum), allows potency assessment [93] and topical application and exhibits metabolic capability. Regarding Episkin, the 3D model is used in the SENS-IS assay based on analysis of two groups of genes: the redox and SENS-IS [94]. Redox group includes genes possessing an antioxidant responsive element (ARE) in their promoter and monitors the redox-protective signals induced through the interaction of sensitizers binding to cysteine amino acids of the Keap1-NRF2 complex [95]; the SENS-IS group includes genes involved in inflammation, danger signals and cell migration to address the complex cascade of events leading to activation of DCs by a sensitizing chemical [96]. Since four dilutions (50%, 10%, 1% and 0.1%) of the test chemical are used, the SENS-IS assay provides potency information predicted according to a threshold value (the minimum test concentration necessary to induce the overexpression of a given number of genes in two groups of genes) [96].

The early phase of skin sensitization (induction of inflammatory cytokines and cytoprotective gene pathways) is evaluated in the epidermal sensitization assay (EpiSensA) by RT-PCR. Initially, the expression of three genes (*ATF3*, *DNAJB4* and *GCLM*) was measured [97]. Finally, the *IL-8* gene was included. Overall sensitivity and accuracy of EpiSensA were relatively higher than those of existing in vitro tests (DPRA, KeratinoSens^TM^ and h-CLAT) [98]. Additionally, in the small-scale study, very good transferability and reproducibility of EpiSensA was stated [99].

**Table 4:** KE2. Methods based on gene expression analysis.

Method	Marker Genes	Model	Dataset	Accuracy [%]	Specificity [%]	Sensitivity [%]	Source /Ref.
				Compared to the LLNA			
EpiSensA	ATF 3; GCLM DNAJB4	3D	16	87.5–100	75–100	83.3–100	[97]
Modified EpiSensA	ATF 3; GCLM DNAJB4; IL-8	3D	72	90	78	94	[98]
SENS-IS	REDOX group: 17 genes, SENS-IS group: 21 genes	3D	150	96.6	95.2	97.7	[96]
HaCaT cell model assay	NQO1, AKR1C2, TXN, IL8, ALDH3A, HMOX1, MafF, GCLC, CYP1A1, MT1, MT2	2D	58	84	92	81	[90]
SenCeeTox	The expression of: eight Nrf2/ARE, one AhR/XRE, two Nrf1/MRE-controlled genes	3D	11	-	-	-	[92]
HaCaT gene signature	*HMOX1*, *STC2* *ADM*, *SRD1*, *cFOS*, *FosL1*, *DNMT3b*, *RBM5*, *CDK12*, *ARD37*	2D	39	96.2	100	95	[91]

The last type of the KE2 methods is based on the evaluation of proinflammatory cytokines (Table 5). Interleukin (IL)-18 plays a key role in the induction of ACD by promoting Th-1-type immune response enhancing the secretion of proinflammatory mediators such as *TNF-a*, *IL-8* and *IFN-c* [100]. Human KC constitutively expresses *IL-18* mRNA and protein, which are induced following the exposure to contact sensitizers [101]. The NCTC 2544, a commercially available epithelial-like skin cell line originating from normal human skin, has been used to identify contact allergens [102] on the basis of *IL-18* production assessed in the cell lysate by the ELISA method. All tested contact sensitizers, including prohaptens, induced a dose-related increase in *IL-18*, whereas both irritants and respiratory allergens failed. The critical point is the use of cells in the appropriate time, between 3 weeks and up to 5 months after thawing [103]. The authors pointed to the possibility of using other than the NCTC 2544 cell line; however, HaCaT gave a large number of false-negative results (19 out of 24 sensitizers were incorrectly identified). The following accuracy, sensitivity and specificity were obtained for 41 substances: 57.2%, 22.2%, 91.7%, respectively [104].

The RhE model derived from normal human keratinocytes has been used in the *IL-18* epidermal equivalent (EE) assay in order to identify contact sensitizers as well as determine sensitizing potency [106]. Several in vitro-reconstructed 3D epidermis models were utilized by the assay. Consistent results were obtained between the different 3D models, and only minor method optimization may be required for different types of commercially available EE and different vehicles. The amount of *IL-18* in culture supernatants after 24 h incubation with the tested chemical was quantified by a commercially available, specific sandwich ELISA. Results are presented as a Stimulation Index (SI), i.e., the ratio of *IL-18* released to the basal *IL-18* found on the epidermis treated with the solvent control. The *IL-18* level is used to discriminate sensitizing from nonsensitizing substances, and cell viability calculated by MTT assay is used to evaluate the potency of the sensitizer. The most optimal prediction model, i.e., ≥5 fold increase in *IL-18* release and viability between 5–40%, showed 95% accuracy. Andres et al. indicated that the critical point for the *IL-18* evaluation is the detection range of the ELISA kit [112]. No results should be extrapolated outside the limits of the calibration standard concentrations. An acceptable *IL-18* release from the nonexposed RhE model was set at 60 ± 40 pg/mL^−1^, which could be used to monitor the model suitability over batches of epidermis. The possibility of using the reconstructed human epidermis *IL-18* assay to assess the sensitizing properties of metal sensitizers was tested. It clearly showed that metal salts fall outside of the applicability domain of the assay due to insufficient amounts of *IL-18* being released and low cytotoxicity [113].

Levels of Il-6 and IL-1α in supernatants after 24 h incubation with tested chemicals were measured by the ELISA method in the HaCaSens assay [114]. Chemical classification is determined using the stimulation index (SI). Nonsensitizers produce SI < 3 in both IL-1α and IL-6, and sensitizers produce SI ≥ 3 in one of the cytokines. HaCaSens obtained 75% sensitivity, 83% specificity and 77% accuracy using 22 coded substances; however, during the validation study, in order to examine transferability, intra- and interlaboratory reproducibility and predictive capacity, it demonstrated a sensitivity of 81.8%, specificity of 87.5% and accuracy of 83.3% in identifying skin sensitizers [107]. The optimized HaCaSens, based on the reduction of doses from four to three, resulted in a sensitivity of 83.3%, a specificity of 80.0% and an accuracy of 81.8% [108].

Based on the Il-1α production by in-house epidermal equivalents (VUMC-EE) and commercial epidermal equivalent (EST1000™ and RHE™), the potency classification can be performed. It is based on the effective chemical concentration required to reduce cell metabolism/viability to 50% of the maximum value (EE-EC50) and the effective chemical concentration required to result in a 10-fold increase in secretion of IL-1α (EE-IL-1α10x value) [111]. The lower the EC50 and EE-IL- 1α10x, the stronger the sensitizing potency. Since this assay does not distinguish sensitizers from nonsensitizers, it should be applied in a two-tiered strategy, i.e., the determination of potency should be conducted following the identification of sensitizers using another method.

The murine cell line HEL-30 was used for assessment of KE2 through evaluation of IL-1α, macrophage inflammatory protein 2 (MIP-2), IL-6 and IL-18 [110]. IL-18 levels did not statistically discriminate between sensitizers and irritants, while IL-6 was not produced by HEL-30; therefore, the combination of IL-1α and MIP-2 gave the most robust result, i.e., overall accuracy: 86% (19 out of 22). Another study using HEL-30 showed a consistent dose–response relationship for IL-1α and IL-18, which is in contrast to the HaCaT cell line, where it was not possible to evaluate three out of four tested chemicals [109].

### 3.3. Key Event 3 (KE3)-Based Methods

KE3 concerns the activation of DCs which are specialized in the processing and presentation of antigen to lymphocytes with the help of a major histocompability complex (MHC). Mainly, the DCs present peptides via class II MCHC recognized by CD4+ T lymphocytes. However, it is possible to present peptides in CD8+ T lymphocytes with MHC I class antigen. Only dendritic cells may present an antigen to naive T lymphocytes (without prior contact with the antigen). After the exposure to hapten, the DCs migrate from the epidermis to local lymph nodes through dermal lymphatic vessels [115] and undergo maturation to become more efficient antigen-presenting cells [116], which results in an increased expression of various cell membrane markers, such as CD40, CD54, CD80, CD83 and CD86 [117], as well as production of the proinflammatory cytokines, e.g., TNFα, IL-6, IL-8 and IL-1β. The summary of the KE3 methods is presented in Table 6.

The following methods adopted by the OECD under the guideline No. 442E allow to distinguish skin sensitizers, i.e., UN GHS category 1, from nonsensitizers:

- Human cell line activation test (h-CLAT);
- U937 cell line activation Test (U-SENS);
- Interleukin-8 Reporter Gene Assay (IL-8 Luc assay);
- Genomic Allergen Rapid Detection (GARD™) for assessment of skin sensitizers (GARD™skin) [118].

The h-CLAT uses the ability of dendritic cells and monocytes to express cell surface markers, i.e., CD86 and CD54, following 24 h of exposure to the test chemical. CD86 and CD54 expression is measured on the human monocytic leukemia cell line (THP-1) derived from the peripheral blood of a male with acute monocytic leukemia [119], which served as a dendritic-cells surrogate [120]. In turn, in the U-SENS method, formerly known as the Myeloid U937 Skin Sensitization Test (MUSST), the human histiocytic lymphoma cell line (U937) is used. This type of cells responds to contact sensitizers by upregulating CD86 expression in a dose-dependent manner after 48 h of treatment [121]. A level of surface markers is measured by flow cytometry, following cell staining with fluorochrome-tagged antibodies. Concurrently, cytotoxicity is measured to assess whether upregulation of surface markers expression occurs at subcytotoxic concentrations. The relative fluorescence intensity (RFI) of surface markers compared to solvent/vehicle control is calculated and used in the prediction model [118]. Due to the possibility of false-negative results for water-insoluble chemicals (especially chemicals with LogKow > 2), the h-CLAT test was modified by applying a different exposure method, i.e., short time exposure to the liquid paraffin (LP) dispersion medium [122]. THP-1 cells shortly (for 5 min) exposed to the test chemicals dispersed and diluted in LP. Then, cells were washed, resuspended in culture medium and incubated for 24 h in the standard conditions (37 °C, 5% CO2). A tiered-system combination of the original h-CLAT and modified h-CLAT provided high sensitivity and accuracy, at 95% and 88%, respectively. However, LP-medium exposure may be unsuitable for prohaptens and LP-insoluble chemicals [122].

Since the regulation EC 1223/2009 includes an animal testing ban for cosmetic products and ingredients, there is a need to replace animal-derived components used in the standard cell culture by nonanimal products. Replacement of fetal bovine serum (FBS) with human serum (HS), bovine serum albumin (BSA) with human serum albumin (HSA) and the use of antibodies derived from a nonanimal source using phage-display (Human Combinatorial Antibody Library; HuCAL) was proposed [123]. The animal-product-free h-CLAT provided appropriate results; all proficiency substances were correctly classified. Currently, authors are pursuing inclusion of the nonanimal modification into the OECD 442E guideline. Other researchers [124] proposed the culturing of THP-1 cells using media free of animal-derived FBS, i.e., RPMI-1640 medium with HL-1™ supplement or X-VIVO™ 10 medium. However, the use of the X-VIVO™ 10 medium required modification of the acceptance criteria for the proficiency assay since THP-1 cells cultured without FBS were more susceptible to hydrodynamic forces, including shear stress during several sample centrifugations and pellet resuspensions. Despite minor adjustments of the original protocol, it was possible to correctly predict the sensitizing potential of ten proficiency substances.

The above methods used cell lines with an extended period of multiplication, mainly from hematopoietic neoplasms, and this approach may have some disadvantages. Cell lines may be characterized by genomic instability and metabolic or signaling deficiency; therefore, primary cells (cord or peripheral blood) have also been used [125]. Plasmocytoid dendritic cells (pDC) obtained from human DCs generated from umbilical cord blood-derived CD34+ progenitor cells may be used for measurement of CD86 due to low CD86 basal expression [126]. Compared to LLNA, the pDC method had sensitivity and specificity of 96% and 86%, respectively. Flow cytometric measurement of CD86 expression after 48 h chemical treatment of the peripheral blood monocyte-derived dendritic cells (PBMDC) was proposed [127]. The application of the PBMDC in the optimized assay (appropriate monocyte isolation procedure, cytokine/chemokine concentrations, treatment conditions and cytotoxicity threshold) allows for the discrimination of sensitizing from nonsensitizing chemicals. The key issue is the initial assessment of CD86 expression; cells with elevated (>50%) CD86 expression are excluded.

A convenient alternative to PBMDC can be U-937 cells. In the U-937 activation test, cells were cultured with the presence of IL-4 to induce the dendritic cell-like phenotype [121]. Then, cells were exposed to test chemicals and analyzed by flow cytometry for CD86 expression and by qRT-PCR for IL-1β and IL-8 gene expressions. A single marker (e.g., CD86, IL-1β or IL-8) was not sufficient for a reliable evaluation of the DC activation; however, a combination of multiple markers allowed a more precise analysis. CD86 surface expression and cell viability is measured at 24 h and 72 h; IL-8 level is measured by ELISA at 72 h only. Gene expression of IL-1β and IL-8 at 24 h should be considered in case of doubtful results.

Not only human cells may be used, but also murine primary cells [128]. After 48 h of incubation of mouse bone marrow-derived dendritic cell (BMDC) with the tested chemicals, changes in MHCII, CD40, CD54 and CD86 expression were evaluated by the flow cytometry. Additionally, a cytokine level in the culture supernatant was evaluated; however, it did not lead to better discrimination of the contact or respiratory allergens. For 20 tested chemicals, a sensitivity of 69% and accuracy of 75% was obtained [129].

The IL-8 Luc assay quantifies changes in IL-8 expression associated with the activation of dendritic cells. This assay uses a stable THP-1-derived IL-8 reporter cell line, THP-G8 characterized by harboring stable luciferase orange (SLO) and stable luciferase red (SLR) genes under the control of IL-8 and glyceraldehyde 3-phosphate dehydrogenase (GAPDH-internal control promotor) promotors, respectively. A quantitative measurement of luciferase gene induction is performed by detecting luminescence. After reaction with firefly D-luciferin, two luciferases emit different colors [118,130]. The mechanism of overproduction of IL-8 by THP-1 cells after exposure to sensitizers is associated with the MAPK (mitogen-activated protein kinase) pathways, and it is strongly induced by subtoxic (60–90% viability) concentration of skin sensitizers [131].

The simultaneous detection of surface antigens and the analysis of cytokines in culture supernatants were also used. A level of IL-1β, IL-8 and TNF-α was analyzed in the culture supernatants of THP-1 cells after 24 h of exposition to the test chemical and expression of two surface markers, i.e., CD54 and CD86. A combination of CD86 expression with IL-8 secretion allowed for the prediction of sensitizers with an accuracy of 95.2% and sensitivity of 93.5% [132].

Using monocyte-derived dendritic cells (moDC) isolated from heparinized leukocyte-enriched buffy coats from different donors by density gradient centrifugation, it was found that the concentration of IL-8 increased after exposure to sensitizers and decreased after exposure to irritants. Therefore, the most promising method for predicting contact allergens seems to be the determination of IL-8 together with the CD83 and CD86 expression [133].

The most recent method introduced into the OECD TG 442E is the genomic allergen rapid detection (GARD), in which the expression of genes in the SenzaCells cell line, which is of human myeloid origin with attributes similar to dendritic cells, is examined. A total of 196 transcripts participating in signaling pathways involved in recognition of foreign substances and DCs maturation is measured (GARDskin Genomic Prediction Signature) [134]. GARD^TM^ skin was validated formally under the supervision of the European Reference Laboratory for Alternatives to Animal Testing (EURL ECVAM); ring study showed an interlaboratory reproducibility of 92.0%, and the cumulative predictive accuracy across the three laboratories was 93.8% [135]. Based on the GARD platform, the prediction of sensitizer potency could be evaluated. The GARD potency gene signature predicts three sensitizer potency classes (categories 1A, 1B and no category) [136]. For this purpose, predictive genomic biomarkers (52 transcripts) were identified using a random forest approach and 70 training samples. The system has been tested on separate chemicals and the balanced accuracies (the average of sensitivity and specificity) of the test are estimated to be 82% for category 1A, 74% for category 1B and 90% for no category, with an overall accuracy of 78%.

A prediction model referred to as VitoSens quantifies the expression of two genes using qPCR, cyclic adenosine monophosphate-responsive element modulator (*CREM*) and monocyte chemotactic protein-1 receptor *(CCR2*) in CD34+ progenitor-derived DCs after 6 h exposure [137]. The VitoSens assay successfully discriminates sensitizing chemicals from nonsensitizing chemicals [138]. The mouse fetal skin-derived dendritic cell line (FSDC) could also be used for the analysis of gene expression and intracellular signaling profiles [139].

**Table 6:** KE3. Summary of methods.

Method	Endpoint	Cell Line	Data Set	Accuracy [%]	Specificity [%]	Sensitivity [%]	Source /Ref.
				Compared to the LLNA			
h-CLAT	expression of CD86/CD54	THP-1	142	85	66	93	[118]
Original h-CLAT + LP h-CLAT	expression of CD86/CD54	THP-1	132	88	70	95	[122]
Animal-free h-CLAT	expression of CD86/CD54	THP-1	10	-	-	-	[123]
Serum free h-CLAT	expression of CD86/CD54	THP-1	10	-	-	-	[124]
U-SENS	expression of CD86	U-937	166	86	65	91	[118]
PBMDC assay	expression of CD86	PBMDC	12	-	-	-	[127]
BMCDs assay	expression of MHC II/CD40/CD54/CD86	BMCD	20	75	-	69	[129]
pDC assay	expression of CD86	pDC	45	91	86	96	[126]
IL-Luc assay	IL-8 level	THP-G8	136	89	53	96	[118]
GARD^TM^skin	gene expression196 transcripts	SenzaCell	75	87.6	89.9	87.2	[118]
GARD potency gene signature	gene expression–52 transcripts	SenzaCell	18	78	-	-	[136]
VitoSens	genes expression: *CREM*, *CCR2*	CD34-DC	73	89	97	82	[137]
FSDC method	gene expression: *Trxr1*, *Hmox1*, *Nqo1* and *Cxcl10* —the p38 MAPK and JNK signalling pathways	FSDC	18	94	100	92	[139]
CD86 and IL-8 release assay	IL-8 level, expression of CD86	THP-1	31	95.2	-	93.5	[132]
U-937 activation test	IL-8 level and expression of CD86	U-937	16	-	-	-	[121]
moDC IL-8 assay	IL-8 level and expression of CD83 and DCD86	moDC	12	-	-	-	[133]
THP-1 IL-8	Il-8 level	THP-1	23	-	-	-	[131]

### 3.4. Key Event 4 (KE4)-Based Methods

Present-day alternative methods for the assessment of skin sensitization focus on KE1–KE3. It is considered that the inclusion of KE4 methods will increase predictive accuracy and reproducibility [140]. The fourth key event is based on the activation and proliferation of T-lymphocytes in the local lymph nodes. This final sensitization phase is evaluated by the local lymph node assays (LLNA) described in the previously mentioned OECD test guidelines 429, 442A and 442B.

A nonanimal alternative for KE4, in vitro human T cell priming assay (hTCPA) is a method which overcomes the main challenges, i.e., small number of naive T cells in the peripheral blood showing specificity for a particular substance and the high activation point of these cells. Peripheral blood mononuclear cells (PBMC) (e.g., from buffy coats or 300–500 mL fresh heparinized blood) from a single, healthy donor are used as a source of moDC and naive T cells. moDCs are incubated overnight with the tested substance in a nontoxic concentration (cell viability ≥ 80%) in the presence of lipopolysaccharides (LPS) or tumor necrosis factor α (TNF-α); as a result, T-cell epitopes are generated. Naive T cells and chemical-modified moDCs are cocultured, optionally in the presence of feeder cells, costimulatory CD28 antibody and cytokines. After 10 days, the IFN-c and TNF-α production by T cells is detected after a rechallenge with chemical-modified moDC [141]. Other researchers [142] evaluated that priming of allergen-specific T cells is limited by several subgroups of immune cells, such as CD1a^neg^ DCs, CD25^+^ T cells and CD56^+^ regulatory cells. The elimination of this subset of cells from peripheral blood lymphocytes (PBLs), can significantly improve T-cell priming to weak sensitizers.

Unlike the hTCPA method, where repeated exposure to a chemical is necessary, other researchers used interleukin-2 expression in T cells to evaluate the KE4. For this purpose, Jurkat cells that express the luciferase gene downstream of the IL-2 promoter (IL-2p) are utilized (IL-2p::Jurkat cells). The test assumes a short treatment of cells with the tested substance (9 h) and requires prior activation by anti-CD3; therefore, memory T cells specific for antigen were probably assessed, and naive T cells were not [140].

The Jurkat Clone E6–1 human T cell line are commonly utilized in in vitro assays on human T-cell activation [143]. Hou et al. used these cells to show early T-cell activation induced by sensitizers using cytometric estimation of CD69 expression. No APCs were involved in this model, which made the procedures much simpler than for the other reported assays. The majority of sensitizing substances increased the expression of the CD69 antigen on the T cells linearly, depending on the concentration of the examined substance.

CD69 RFI ≥ 1.5 was determined to be the positive criterion for skin sensitizer classification when cell viability ≥ 50%. This method showed a good predictivity for skin sensitizers; the sensitivity (79.4%), specificity (88.9%) and accuracy (82.7%) were obtained for the 52 tested reference chemicals [144].

### 3.5. Coculture Methods

Different types of cells, including primary as well as immortalized cell lines, are used in coculture methods. The 2D coculture methods developed in recent years mainly use keratinocytes cell lines [145]. Some researchers use the HaCaT cell line [146,147,148,149,150,151]; however, the NCTC 2544 cell line is also used [152,153]. For dendritic cells, cell lines such as THP-1 [153,154,155,156] or MUTZ-3 are used. The advantage of MUTZ-3 cells is their physiological similarity to dendritic cells [157], and these cells are commonly used to obtain Langerhans cells (MUTZ-LC) or dendritic cells (MUTZ-DC) generated under a cytokine cocktail. Since usage of immortalized cells may be associated with inconsistent results due to the possibility of genotypic and phenotypic variability of cells at high passages, impaired signaling mechanisms or reduced metabolic activity [125,158], primary cells are also used in coculture methods. Primary keratinocytes have been used in indirect coculture [155,159] and direct coculture with PBMC [148]. In the recently presented coculture method, primary keratinocytes seeded into inserts were used to keratinize, i.e., create a multilayered structure [156]. In the case of dendritic cells, the most commonly used are DC derived from blood cell progenitors [125], mainly from CD14 + monocytes derived from peripheral blood or hematopoietic CD34 + progenitor cells derived from peripheral blood, umbilical cord blood or bone marrow [127,133,137].

Coculture endpoints are similar to monoculture methods, e.g., analysis of surface antigens or inflammatory cytokines. It should be noted that the interaction of two or more cell types may significantly affect the magnitude of the cellular response [160]. The communication between keratinocytes and dendritic cells is a two-way reaction. On the one hand, THP-1 cells stimulate keratinocytes to secrete cytokines; on the other hand, keratinocytes increase the activation of dendritic cells in response to sensitizing substances. Ohtani et al. showed that the adenosine triphosphate (ATP) released by keratinocytes acts synergistically with hapten, causing the maturation of dendritic cells [161], which may contribute to more effective detection of weak and moderate allergens [152]. It has also been shown that the induction of CD54 and CD86 antigens on THP-1 cells already occurs at an almost nontoxic concentrations in the case of coculture [155], when subcytotoxic concentrations are necessary for the induction of these antigens in the monoculture [162,163]. The chance to obtain a positive reaction at lower concentrations of tested substance may increase the possibility of testing poorly water-soluble substances.

The great advantage of coculture methods seems to be the increased possibility of detecting prohaptens, thanks to the metabolic activity of keratinocytes [152,154,155] and the use of more complex coculture models containing, e.g., fibroblasts [149].

Many coculture methods use reconstructed human epidermis models (RhE) in conjunction with dendritic cells in a common medium or immunocompetent skin equiva-lents (SE). Such models provide the possibility of topical exposure and reflect the in vivo skin functions to a greater extent [164,165].

The development of coculture methods involves considering many aspects, includ-ing the type of cells (primary cells, cell lines), the format of the coculture (direct, indirect) and the selection of endpoints [145]. The exposure time of the coculture system to test substances is also essential and should be the subject of extensive research [150].

## 4. Conclusions

Assessing skin sensitization is mandatory not only for chemicals but also for substances used in cosmetic products. Regulatory authorities in accordance with the 3Rs principle introduced by W.M.S. Russel and R.L. Burch in 1959 [166] promote the replacement of in vivo research with alternative methods in the field of skin-sensitization assessment as well.

At the moment, it is not possible to replace the evaluation of sensitization with animals using one alternative method to achieve an adequate level of hazard identification. In contrast to LLNA, in vitro/in chemico methods are not suitable for classification of potency, i.e., subcategory 1A and 1B, according to UN GHS when it is used on its own. To overcome this limitation, an attempt of combining different sources of information (in silico, in chemico, in vitro) was made in order to attain predictive ability at the appropriate level to predict responses in humans [27]. The assessment of sensitization potential within DAs and IATAs is mainly based on methods concerning KE1-KE3 of AOP skin sensitization. Additional data on the fourth KE may complement the information from KE1-KE3 as a part of DAs and IATAs and contribute to increasing the prognostic capacity [144].

Alternative methods have limitations that must be considered in order to obtain acceptable results under the conditions of the method. Limitations may arise from the nature of the test system, the properties of the analyzed substance and the use of appropriate threshold values or predictive models, and there may be technical limitations.

The solubility of the tested material is one of the basic issues limiting the use of a given method, since methods require the use of a specific solvent and concentration. Especially, cell-based methods require aqueous conditions that could exclude the analysis of strongly lipophilic substances. On the other hand, in chemico methods may require specific reaction conditions, e.g., strongly acidic or alkaline conditions, which may limit the possibility of examination of unstable substances, or precipitate in such pH conditions. Some methods require the use of a molar concentration, which makes it impossible to analyze, i.e., mixtures of unknown composition. The gravimetric approach may allow the analysis of mixtures, which is particularly important in the case of cosmetics ingredients that cannot be tested by in vivo methods in the EU. Other methods allow the use of a different concentration, e.g., a lower one, but then, only a positive result is considered, as a negative result is not conclusive. The use of an appropriately high concentration may also be limited in cell-based methods by high cytotoxicity.

The detection of prohaptens (S-9 fraction, microsomes fraction). Although Natsch et al. report that a small fraction of sensitizing substances requires metabolic activation [87], exogenous metabolic systems are incorporated in alternative methods to apply a test system characterized by metabolic capability, e.g., the reconstructed human epidermis (RhE).

A selection of the appropriate threshold value or the prediction model providing acceptable performance of the method could be challenging.

Limitations of alternative methods contribute to their improvement as well as to the search for new opportunities. The market demand or legislation also contribute to the development of alternative methods. There is a need to adapt existing methods or develop new ones to assess sensitization potential for ingredients of cosmetics, nanomaterials or medical devices.

Development directions may be different, i.e., searching for the most efficient research system among primary or continuous cell lines but also 3D models such as equivalents of the human epidermis or skin. Coculture methods may also be promising. A combination of the main cells involved in the sensitization process, such as keratinocytes and dendritic cells, reflects the mechanisms taking place in vivo, taking into account the intercellular interactions and, in complex methods, the influence of the microenvironment on the modulation of the allergic reaction [140,145].

The continuous progress in knowledge underlying skin-sensitization mechanisms is the basis for the development of alternative methods as well as DAs/IATA [167]. However, further refinement of these methods is still needed to achieve better correlation with human data.

## Figures and Tables

**Table 1:** Characteristic of DAs under OECD TG 497 [34,35].

Type of DA	Types of Covered KE	Methods	Prediction
2o3 DA	KE1, KE2, KE3	DPRA, KeratinoSensTM, h-CLAT	Hazard
ITSv1	KE1, KE3	DPRA, h-CLAT, DEREK Nexus v6.1.0	Hazard, potency
ITSv2	KE1, KE3	DPRA, h-CLAT, OECD QSAR Toolbox v4.5	Hazard, potency

**Table 5:** KE2. Methods based on the evaluation of proinflammatory cytokines.

Method	Cytokine	Test System	Dataset	Accuracy [%]	Specificity [%]	Sensitivity [%]	Source/ Ref.
				Compared to the LLNA			
NCTC 2544 IL-18 assay	IL-18	2D/ NCTC 2544	33	97	94.1	100	[105]
HaCaT IL-18 assay	IL-18	2D/HaCaT	41 *	57.2	91.7	22.2	[104]
IL-18 EE potency assay	IL-18	3D/ RhE	27	95	-	-	[106]
HaCaSens	IL-1α, IL-6	2D/HaCaT	20	83.3	87.5	81.8	[107]
Optimized HaCaSens	IL-1α IL-6	2D/HaCaT	22	81.8	80	83.3	[108]
HaCaT/HEL30 assay	IL-1α IL-18	2D/ HEL-30/HaCaT	4	-	-	-	[109]
HEL-30 IL-1α, MIP-2	IL-1α MIP-2	2D/HEL-30	22	86	-	-	[110]
IL-1α EE potency assay	IL-1α	3D/RhE	16	-	-	-	[111]

* Compared to the human data.

## Data Availability

Not applicable.

## References

[1] Peiser M., Tralau T., Heidler J., Api A.M., Arts J.H.E., Basketter D.A., English J., Diepgen T.L., Fuhlbrigge R.C., Gaspari A.A. (2012). Allergic contact dermatitis: Epidemiology, molecular mechanisms, in vitro methods and regulatory aspects. Cell. Mol. Life Sci..

[2] Thyssen J.P., Linneberg A., Menné T., Johansen J.D. (2007). The epidemiology of contact allergy in the general population-prevalence and main findings. Contact Dermat..

[3] Clouet E., Kerdine-Römer S., Ferret P.-J. (2017). Comparison and validation of an in vitro skin sensitization strategy using a data set of 33 chemical references. Toxicol. Vitr..

[4] Caloni F., De Angelis I., Hartung T. (2022). Replacement of animal testing by integrated approaches to testing and assessment (IATA): A call for in vivitrosi. Arch. Toxicol..

[5] OECD (2009). Test No. 437: Bovine Corneal Opacity and Permeability Test Method for Identifying Ocular Corrosives and Severe Irritants [Internet]. https://www.oecd-ilibrary.org/content/publication/9789264076303-en.

[6] OECD (2018). Test No. 438: Isolated Chicken Eye Test Method for Identifying (i) Chemicals Inducing Serious Eye Damage and (ii) Chemicals Not Requiring Classification for Eye Irritation or Serious Eye Damage [Internet]. https://www.oecd-ilibrary.org/content/publication/9789264203860-en.

[7] OECD (2015). Test No. 430: In Vitro Skin Corrosion: Transcutaneous Electrical Resistance Test Method (TER) [Internet]. https://www.oecd-ilibrary.org/content/publication/9789264242739-en.

[8] Wick P., Chortarea S., Guenat O.T., Roesslein M., Stucki J.D., Hirn S., Fink A., Rothen-Rutishauser B. (2015). In Vitro-ex vivo model systems for nanosafety assessment. Eur. J. Nanomed..

[9] Kandárová H., Letašiová S. (2011). Alternative methods in toxicology: Pre-validated and validated methods. Interdiscip. Toxicol..

[10] Raies A.B., Bajic V.B. (2016). In silico toxicology: Computational methods for the prediction of chemical toxicity. Wiley Interdiscip. Rev. Comput. Mol. Sci..

[11] Hemmerich J., Ecker G.F. (2020). In silico toxicology: From structure–activity relationships towards deep learning and adverse outcome pathways. WIREs Comput. Mol. Sci..

[12] Kim J.Y., Kim M.K., Kim K.B., Kim H.S., Lee B.M. (2019). Quantitative structure-activity and quantitative structure-property relationship approaches as alternative skin sensitization risk assessment methods. J. Toxicol. Environ. Health A..

[13] De Ávila R.I., Lindstedt M., Valadares M.C. (2019). The 21st Century movement within the area of skin sensitization assessment: From the animal context towards current human-relevant in vitro solutions. Regul. Toxicol. Pharmacol..

[14] Hartung T. (2011). From alternative methods to a new toxicology. Eur. J. Pharm. Biopharm..

[15] Maertens A., Golden E., Luechtefeld T.H., Hoffmann S., Tsaioun K., Hartung T. (2022). Probabilistic risk assessment-the keystone for the future of toxicology. ALTEX.

[16] Bos J.D., Meinardi M.M.H.M. (2000). The 500 Dalton rule for the skin penetration of chemical compounds and drugs. Exp. Dermatol..

[17] Kimber I. (2019). The activity of methacrylate esters in skin sensitisation test methods: A review. Regul. Toxicol. Pharmacol..

[18] Roberts D.W., Api A.M., Aptula A.O. (2016). Chemical applicability domain of the Local Lymph Node Assay (LLNA) for skin sensitisation potency. Part 2. The biological variability of the murine Local Lymph Node Assay (LLNA) for skin sensitisation. Regul. Toxicol. Pharmacol..

[19] Martin S.F., Rustemeyer T., Thyssen J.P. (2018). Recent advances in understanding and managing contact dermatitis. F1000Reserach.

[20] Martins L.E.A.M., Dos Reis V.M.S. (2011). Immunopathology of allergic contact dermatitis. An. Bras. De Dermatol..

[21] Aptula A.O., Roberts D.W., Pease C.K. (2007). Haptens, prohaptens and prehaptens, or electrophiles and proelectrophiles. Contact Dermat..

[22] Urbisch D., Becker M., Honarvar N., Kolle S.N., Mehling A., Teubner W., Wareing B., Landsiedel R. (2016). Assessment of Pre- and Pro-haptens Using Nonanimal Test Methods for Skin Sensitization. Chem. Res. Toxicol.

[23] Karlberg A.-T., Börje A., Duus Johansen J., Lidén C., Rastogi S., Roberts D., Uter W., White I.R. (2013). Activation of non-sensitizing or low-sensitizing fragrance substances into potent sensitizers-prehaptens and prohaptens. Contact Dermat..

[24] Gądarowska D., Krakowian D., Daniel-Wójcik A., Mrzyk I., Młynarczuk-Biały I., Biały Ł. (2021). Co-culture methods in the skin sensitization testing-review. Advances in Biomedical Research-Cancer and Miscellaneous.

[25] Kimber I., Basketter D.A., Gerberick G.F., Dearman R.J. (2002). Allergic contact dermatitis. Int. Immunopharmacol..

[26] OECD (2022). Test No. 406: Skin Sensitisation [Internet]. https://www.oecd-ilibrary.org/content/publication/9789264070660-en.

[27] OECD (2010). Test No. 429: Skin Sensitisation [Internet]. https://www.oecd-ilibrary.org/content/publication/9789264071100-en.

[28] OECD (2010). Test No. 442A: Skin Sensitization [Internet]. https://www.oecd-ilibrary.org/content/publication/9789264090972-en.

[29] OECD (2018). Test No. 442B: Skin Sensitization [Internet]. https://www.oecd-ilibrary.org/content/publication/9789264090996-en.

[30] Gwaltney-Brant S., Gupta R.C. (2014). Chapter 22-Immunotoxicity biomarkers. Biomarkers in Toxicology.

[31] Williams W.C., Copeland C., Boykin E., Quell S.J., Lehmann D.M. (2015). Development and utilization of an ex vivo bromodeoxyuridine local lymph node assay protocol for assessing potential chemical sensitizers. J. Appl. Toxicol..

[32] UE (2016). Commission regulation (EU) 2016/1688 of 20 September 2016 amending Annex VII to Regulation (EC) No 1907/2006 of the European Parliament and of the council on the Registration. Off. J. Eur. Communities.

[33] Casati S., Aschberger K., Barroso J., Casey W., Delgado I., Kim T.S., Kleinstreuer N., Kojima H., Lee J.K., Lowit A. (2018). Standardisation of defined approaches for skin sensitisation testing to support regulatory use and international adoption: Position of the International Cooperation on Alternative Test Methods. Arch. Toxicol..

[34] OECD (2021). Guideline No. 497: Defined Approaches on Skin Sensitisation [Internet]. https://www.oecd-ilibrary.org/content/publication/b92879a4-en.

[35] Basketter D.A., Gerberick G.F. (2022). Skin Sensitization Testing: The Ascendancy of Non-Animal Methods. Cosmetics.

[36] Wilm A., Kühnl J., Kirchmair J. (2018). Computational approaches for skin sensitization prediction. Crit. Rev. Toxicol..

[37] Strickland J., Truax J., Corvaro M., Settivari R., Henriquez J., McFadden J., Gulledge T., Johnson V., Gehen S., Germolec D. (2022). Application of Defined Approaches for Skin Sensitization to Agrochemical Products. Front. Toxicol..

[38] Reynolds J., MacKay C., Gilmour N., Miguel-Vilumbrales D., Maxwell G. (2019). Probabilistic prediction of human skin sensitiser potency for use in next generation risk assessment. Comput. Toxicol..

[39] Kolle S.N., Hill E., Raabe H., Landsiedel R., Curren R. (2019). Regarding the references for reference chemicals of alternative methods. Toxicol. Vitr..

[40] Kleinstreuer N.C., Hoffmann S., Alépée N., Allen D., Ashikaga T., Casey W., Clouet E., Cluzel M., Desprez B., Gellatly N. (2018). Non-animal methods to predict skin sensitization (II): An assessment of defined approaches. Crit. Rev. Toxicol..

[41] Natsch A., Landsiedel R., Kolle S.N. (2021). A triangular approach for the validation of new approach methods for skin sensitization. ALTEX.

[42] Gilmour N., Kimber I., Williams J., Maxwell G. (2019). Skin sensitization: Uncertainties, challenges, and opportunities for improved risk assessment. Contact Dermat..

[43] Daniel A.B., Strickland J., Allen D., Casati S., Zuang V., Barroso J., Whelan M., Régimbald-Krnel M., Kojima H., Nishikawa A. (2018). International regulatory requirements for skin sensitization testing. Regul. Toxicol. Pharmacol..

[44] OECD (2022). Test No. 442C: In Chemico Skin Sensitisation [Internet]. https://www.oecd-ilibrary.org/content/publication/9789264229709-en.

[45] Yamamoto Y., Tahara H., Usami R., Kasahara T., Jimbo Y., Hioki T., Fujita M. (2015). A novel in chemico method to detect skin sensitizers in highly diluted reaction conditions. J. Appl. Toxicol..

[46] Natsch A., Gfeller H. (2008). LC-MS–Based Characterization of the Peptide Reactivity of Chemicals to Improve the In Vitro Prediction of the Skin Sensitization Potential. Toxicol. Sci..

[47] Fujita M., Yamamoto Y., Tahara H., Kasahara T., Jimbo Y., Hioki T. (2014). Development of a prediction method for skin sensitization using novel cysteine and lysine derivatives. J. Pharmacol. Toxicol. Methods.

[48] Akimoto M., Yamamoto Y., Watanabe S., Yamaga H., Yoshida K., Wakabayashi K., Tahara Y., Horie N., Fujimoto K., Kusakari K. (2020). Oxidation of a cysteine-derived nucleophilic reagent by dimethyl sulfoxide in the amino acid derivative reactivity assay. J. Appl. Toxicol..

[49] Yamamoto Y., Fujita M., Wanibuchi S., Katsuoka Y., Ono A., Kasahara T. (2019). Expanding the applicability of the amino acid derivative reactivity assay: Determining a weight for preparation of test chemical solutions that yield a predictive capacity identical to the conventional method using molar concentration and demonstrating the capacity to detect sensitizers in liquid mixtures. J. Pharmacol. Toxicol. Methods.

[50] Fujita M., Yamamoto Y., Wanibuchi S., Katsuoka Y., Kasahara T. (2019). The underlying factors that explain why nucleophilic reagents rarely co-elute with test chemicals in the ADRA. J. Pharmacol. Toxicol. Methods.

[51] Fujita M., Yamamoto Y., Watanabe S., Sugawara T., Wakabayashi K., Tahara Y., Horie N., Fujimoto K., Kusakari K., Kurokawa Y. (2018). Cause of and countermeasures for oxidation of the cysteine-derived reagent used in the amino acid derivative reactivity assay. J. Appl. Toxicol..

[52] Fujita M., Yamamoto Y., Wanibuchi S., Katsuoka Y., Kasahara T. (2019). A newly developed means of HPLC-fluorescence analysis for predicting the skin sensitization potential of multi-constituent substances using ADRA. Toxicol. Vitr..

[53] Sanderson P.N., Simpson W., Cubberley R., Aleksic M., Gutsell S., Russell P.J. (2016). Mechanistic understanding of molecular initiating events (MIEs) using NMR spectroscopy. Toxicol. Res..

[54] Chittiboyina A.G., Avonto C., Rua D., Khan I.A. (2015). Alternative Testing Methods for Skin Sensitization: NMR Spectroscopy for Probing the Reactivity and Classification of Potential Skin Sensitizers. Chem. Res. Toxicol..

[55] Avonto C., Chittiboyina A., Rua D., A Khan I. (2015). A fluorescence high throughput screening method for the detection of reactive electrophiles as potential skin sensitizers. Toxicol. Appl. Pharmacol..

[56] Avonto C., Wang Y.-H., Chittiboyina A.G., Vukmanovic S., Khan I.A. (2019). In chemico assessment of potential sensitizers: Stability and direct peptide reactivity of 24 fragrance ingredients. J. Appl. Toxicol..

[57] Avonto C., Chittiboyina A.G., Sadrieh N., Vukmanovic S., Khan I.A. (2018). In chemico skin sensitization risk assessment of botanical ingredients. J. Appl. Toxicol..

[58] Zhang F., Erskine T., Klapacz J., Settivari R., Marty S. (2018). A highly sensitive and selective high pressure liquid chromatography with tandem mass spectrometry (HPLC/MS-MS) method for the direct peptide reactivity assay (DPRA). J. Pharmacol. Toxicol. Methods.

[59] Jeong Y.H., An S., Shin K., Lee T.R. (2013). Peptide reactivity assay using spectrophotometric method for high-throughput screening of skin sensitization potential of chemical haptens. Toxicol. Vitr..

[60] Cho S.-A., Jeong Y.H., Kim J.H., Kim S., Cho J.-C., Heo Y., Suh K.-D., An S., Shin K. (2014). Method for detecting the reactivity of chemicals towards peptides as an alternative test method for assessing skin sensitization potential. Toxicol. Lett..

[61] Cho S.-A., An S., Park J.-H. (2019). High-throughput screening (HTS)-based spectrophotometric direct peptide reactivity assay (Spectro-DPRA) to predict human skin sensitization potential. Toxicol. Lett..

[62] Nepal M.R., Shakya R., Kang M.J., Jeong T.C. (2018). A simple in chemico method for testing skin sensitizing potential of chemicals using small endogenous molecules. Toxicol. Lett..

[63] Gerberick G.F., Troutman A.J., Foertsch M.L., Vassallo D.J., Quijano M., Dobson L.M.R., Goebel C., Lepoittevin J.-P. (2009). Investigation of Peptide Reactivity of Pro-hapten Skin Sensitizers Using a Peroxidase-Peroxide Oxidation System. Toxicol. Sci..

[64] Lalko J.F., Dearman R.J., Gerberick G.F., Troutman J.A., Api A.M., Kimber I. (2013). Reactivity of chemical respiratory allergens in the Peroxidase Peptide Reactivity Assay. Toxicol. Vitr..

[65] Bauer B., Andersson S.I., Stenfeldt A.-L., Simonsson C., Bergström J., Ericson M.B., Jonsson C.A., Broo K.S. (2011). Modification and Expulsion of Keratins by Human Epidermal Keratinocytes upon Hapten Exposure in Vitro. Chem. Res. Toxicol..

[66] Dietz L., Kinzebach S., Ohnesorge S., Franke B., Goette I., Koenig-Gressel D., Thierse H.J. (2013). Proteomic allergen–peptide/protein interaction assay for the identification of human skin sensitizers. Toxicol. Vitr..

[67] Chipinda I., Mbiya W., Adigun R.A., Morakinyo M.K., Law B.F., Simoyi R.H., Siegel P.D. (2014). Pyridoxylamine reactivity kinetics as an amine based nucleophile for screening electrophilic dermal sensitizers. Toxicology.

[68] Chipinda I., Ajibola R.O., Morakinyo M.K., Ruwona T.B., Simoyi R.H., Siegel P.D. (2010). Rapid and simple kinetics screening assay for electrophilic dermal sensitizers using nitrobenzenethiol. Chem. Res. Toxicol..

[69] Petersen E.J., Uhl R., Toman B., Elliott J.T., Strickland J., Truax J., Gordon J. (2022). Development of a 96-Well Electrophilic Allergen Screening Assay for Skin Sensitization Using a Measurement Science Approach. Toxics.

[70] Suzuki M., Hirota M., Hagino S., Itagaki H., Aiba S. (2009). Evaluation of changes of cell-surface thiols as a new biomarker for in vitro sensitization test. Toxicol. Vitr..

[71] Imai N., Takeyoshi M., Aizawa S., Tsurumaki M., Kurosawa M., Toyoda A., Sugiyama M., Kasahara K., Ogata S., Omori T. (2022). Improved performance of the SH test as an in vitro skin sensitization test with a new predictive model and decision tree. J. Appl. Toxicol..

[72] Gusev E., Zhuravleva Y. (2022). Inflammation: A New Look at an Old Problem. Int. J. Mol. Sci..

[73] Helou D.G., Martin S.F., Pallardy M., Chollet-Martin S., Kerdine-Römer S. (2019). Nrf2 Involvement in Chemical-Induced Skin Innate Immunity. Front. Immunol..

[74] Dinkova-Kostova A.T., Kostov R.V., Canning P. (2017). Keap1, the cysteine-based mammalian intracellular sensor for electrophiles and oxidants. Arch. Biochem. Biophys..

[75] Natsch A. (2009). The Nrf2-Keap1-ARE Toxicity Pathway as a Cellular Sensor for Skin Sensitizers—Functional Relevance and a Hypothesis on Innate Reactions to Skin Sensitizers. Toxicol. Sci..

[76] Emter R., Ellis G., Natsch A. (2010). Performance of a novel keratinocyte-based reporter cell line to screen skin sensitizers In Vitro. Toxicol. Appl. Pharmacol..

[77] Dinkova-Kostova A.T., Holtzclaw W.D., Kensler T.W. (2005). The Role of Keap1 in Cellular Protective Responses. Chem. Res. Toxicol..

[78] Kansanen E., Kuosmanen S.M., Leinonen H., Levonen A.-L. (2013). The Keap1-Nrf2 pathway: Mechanisms of activation and dysregulation in cancer. Redox Biol..

[79] OECD (2022). Test No. 442D: In Vitro Skin Sensitisation [Internet]. https://www.oecd-ilibrary.org/content/publication/9789264229822-en.

[80] Ramirez T., Mehling A., Kolle S.N., Wruck C.J., Teubner W., Eltze T., Aumann A., Urbisch D., van Ravenzwaay B., Landsiedel R. (2014). LuSens: A keratinocyte based ARE reporter gene assay for use in integrated testing strategies for skin sensitization hazard identification. Toxicol. Vitr..

[81] Emter R., Natsch A. (2015). A fast Resazurin-based live viability assay is equivalent to the MTT-test in the KeratinoSens assay. Toxicol. Vitr..

[82] Uibel F., Mühleisen A., Köhle C., Weimer M., Stummann T., Bremer S., Schwarz M. (2009). ReProGlo: A new stem cell-based reporter assay aimed to predict embryotoxic potential of drugs and chemicals. Reprod. Toxicol..

[83] Settivari R.S., Gehen S.C., Amado R.A., Visconti N.R., Boverhof D.R., Carney E.W. (2015). Application of the KeratinoSensTM assay for assessing the skin sensitization potential of agrochemical active ingredients and formulations. Regul. Toxicol. Pharmacol..

[84] Andres E., Sá-Rocha V.M., Barrichello C., Haupt T., Ellis G., Natsch A. (2013). The sensitivity of the KeratinoSensTM assay to evaluate plant extracts: A pilot study. Toxicol. Vitr..

[85] Nishijo T., Miyazawa M., Saito K., Otsubo Y., Mizumachi H., Sakaguchi H. (2019). Sensitivity of KeratinoSensTM and h-CLAT for detecting minute amounts of sensitizers to evaluate botanical extract. J. Toxicol. Sci..

[86] Mertl E., Riegel E., Glück N., Ettenberger-Bornberg G., Lin G., Auer S., Haller M., Wlodarczyk A., Steurer C., Kirchnawy C. (2019). A dual luciferase assay for evaluation of skin sensitizing potential of medical devices. Mol. Biol. Rep..

[87] Natsch A., Haupt T. (2013). Utility of Rat Liver S9 Fractions to Study Skin-Sensitizing Prohaptens in a Modified KeratinoSens Assay. Toxicol. Sci..

[88] Huth L., Moss E., Huth S., Skazik C., Karlberg A., Lepoittevin J., Baron J., Merk H. (2017). 429 Prohapten-activation by human cutaneous cytochrome P450 isoenzymes—Identified with a modified KeratinoSens assay. J. Investig. Dermatol..

[89] Natsch A., Emter R. (2007). Skin Sensitizers Induce Antioxidant Response Element Dependent Genes: Application to the In Vitro Testing of the Sensitization Potential of Chemicals. Toxicol. Sci..

[90] McKim J.M., Keller D.J., Gorski J.R. (2010). A new in vitro method for identifying chemical sensitizers combining peptide binding with ARE/EpRE-mediated gene expression in human skin cells. Cutan. Ocul. Toxicol..

[91] van der Veen J.W., Pronk T.E., van Loveren H., Ezendam J. (2013). Applicability of a keratinocyte gene signature to predict skin sensitizing potential. Toxicol. Vitr..

[92] McKim J.M., Keller D.J., Gorski J.R. (2012). An in vitro method for detecting chemical sensitization using human reconstructed skin models and its applicability to cosmetic, pharmaceutical, and medical device safety testing. Cutan. Ocul. Toxicol..

[93] Galbiati V., Gibbs S., Roggen E., Corsini E. (2018). Development of an In Vitro Method to Estimate the Sensitization Induction Level of Contact Allergens. Curr. Protoc. Toxicol..

[94] Cottrez F., Boitel E., Auriault C., Aeby P., Groux H. (2015). Genes specifically modulated in sensitized skins allow the detection of sensitizers in a reconstructed human skin model. Development of the SENS-IS assay. Toxicol.Vitr. Int. J. Publ. Assoc. BIBRA.

[95] Uruno A., Motohashi H. (2011). The Keap1–Nrf2 system as an in vivo sensor for electrophiles. Nitric. Oxide.

[96] Cottrez F., Boitel E., Ourlin J.-C., Peiffer J.-L., Fabre I., Henaoui I.-S., Mari B., Vallauri A., Paquet A., Barbry P. (2016). SENS-IS, a 3D reconstituted epidermis based model for quantifying chemical sensitization potency: Reproducibility and predictivity results from an inter-laboratory study. Toxicol. Vitr..

[97] Saito K., Nukada Y., Takenouchi O., Miyazawa M., Sakaguchi H., Nishiyama N. (2013). Development of a new in vitro skin sensitization assay (Epidermal Sensitization Assay; EpiSensA) using reconstructed human epidermis. Toxicol. Vitr..

[98] Saito K., Takenouchi O., Nukada Y., Miyazawa M., Sakaguchi H. (2017). An in vitro skin sensitization assay termed EpiSensA for broad sets of chemicals including lipophilic chemicals and pre/pro-haptens. Toxicol. Vitr..

[99] Mizumachi H., Sakuma M., Ikezumi M., Saito K., Takeyoshi M., Imai N., Okutomi H., Umetsu A., Motohashi H., Watanabe M. (2018). Transferability and within- and between-laboratory reproducibilities of EpiSensA for predicting skin sensitization potential in vitro: A ring study in three laboratories. J. Appl. Toxicol..

[100] Cumberbatch M., Dearman R.J., Antonopoulos C., Groves R.W., Kimber I. (2001). Interleukin (IL)-18 induces Langerhans cell migration by a tumour necrosis factor-alpha- and IL-1beta-dependent mechanism. Immunology.

[101] Naik S.M., Cannon G., Burbach G.J., Singh S.R., Swerlick R.A., Ansel J.C., Caughman S.W., Wilcox J.N. (1999). Human Keratinocytes Constitutively Express Interleukin-18 and Secrete Biologically Active Interleukin-18 After Treatment with Pro-Inflammatory Mediators and Dinitrochlorobenzene. J. Investig. Dermatol..

[102] Corsini E., Mitjans M., Galbiati V., Lucchi L., Galli C., Marinovich M. (2009). Use of IL-18 production in a human keratinocyte cell line to discriminate contact sensitizers from irritants and low molecular weight respiratory allergens. Toxicol. Vitr..

[103] Corsini E., Galbiati V., Mitjans M., Galli C., Marinovich M. (2013). NCTC 2544 and IL-18 production: A tool for the identification of contact allergens. Toxicol. Vitr..

[104] Van der Veen J.W., Rorije E., Emter R., Natsch A., van Loveren H., Ezendam J. (2014). Evaluating the performance of integrated approaches for hazard identification of skin sensitizing chemicals. Regul. Toxicol. Pharmacol..

[105] Galbiati V., Corsini E. (2012). The NCTC 2544 IL-18 Assay for the In Vitro Identification of Contact Allergens. Curr. Protoc. Toxicol..

[106] Gibbs S., Corsini E., Spiekstra S.W., Galbiati V., Fuchs H.W., Degeorge G., Troese M., Hayden P., Deng W., Roggen E. (2013). An epidermal equivalent assay for identification and ranking potency of contact sensitizers. Toxicol. Appl. Pharmacol..

[107] Chung H., Quan H., Jung D., Ravi G., Cho A., Kang M.J., Kim E., Che J.-H., Lee E.-S., Jeong T.C. (2018). Intra- and inter-laboratory reproducibility and predictivity of the HaCaSens assay: A skin sensitization test using human keratinocytes, HaCaT. Toxicol. Vitr..

[108] Jeon B., Kim M.O., Kim Y., Han H., Yun J.-H., Kim J., Huang Y., Choi Y., Cho C.-H., Kang B.-C. (2019). Optimization and validation of a method to identify skin sensitization hazards using IL-1 α and IL-6 secretion from HaCaT. Toxicol. Vitr..

[109] Och F.M.M.V., Loveren H.V., Wolfswinkel J.C.V., Machielsen A.J.C., Vandebriel R.J. (2005). Assessment of potency of allergenic activity of low molecular weight compounds based on IL-1α and IL-18 production by a murine and human keratinocyte cell line. Toxicology.

[110] Son D., Na Y., Cho W.-S., Lee B.-H., Heo Y., Park J.-H., Seok S.H. (2013). Differentiation of skin sensitizers from irritant chemicals by interleukin-1α and macrophage inflammatory protein-2 in murine keratinocytes. Toxicol. Lett..

[111] dos Santos G.G., Spiekstra S.W., Sampat-Sardjoepersad S.C., Reinders J., Scheper R.J., Gibbs S. (2011). A potential in vitro epidermal equivalent assay to determine sensitizer potency. Toxicol. Vitr..

[112] Andres E., Barry M., Hundt A., Dini C., Corsini E., Gibbs S., Roggen E.L., Ferret P. (2017). Preliminary performance data of the RHE/IL-18 assay performed on SkinEthicTM RHE for the identification of contact sensitizers. Int. J. Cosmet. Sci..

[113] Gibbs S., Kosten I., Veldhuizen R., Spiekstra S., Corsini E., Roggen E., Rustemeyer T., Feilzer A.J., Kleverlaan C.J. (2018). Assessment of metal sensitizer potency with the reconstructed human epidermis IL-18 assay. Toxicology.

[114] Jung D., Che J.-H., Lim K.-M., Chun Y.-J., Heo Y., Seok S.H. (2016). Discrimination of skin sensitizers from non-sensitizers by interleukin-1α and interleukin-6 production on cultured human keratinocytes. J. Appl. Toxicol..

[115] Lukas M., Stössel H., Hefel L., Imamura S., Fritsch P., Sepp N.T., Schuler G., Romani N. (1996). Human Cutaneous Dendritic Cells Migrate Through Dermal Lymphatic Vessels in a Skin Organ Culture Model. J. Investig. Dermatol..

[116] Gober M.D., Gaspari A.A. (2008). Allergic Contact Dermatitis. Dermatol. Immun..

[117] Stępnik M., Arkusz J. (2003). Molecular events associated with dendritic cells activation by contact sensitizers. Int. J. Occup. Med. Environ. Health.

[118] OECD (2022). Test No. 442E: In Vitro Skin Sensitisation [Internet]. https://www.oecd-ilibrary.org/content/publication/9789264264359-en.

[119] Tsuchiya S., Yamabe M., Yamaguchi Y., Kobayashi Y., Konno T., Tada K. (1980). Establishment and characterization of a human acute monocytic leukemia cell line (THP-1). Int. J. Cancer.

[120] Ashikaga T., Yoshida Y., Hirota M., Yoneyama K., Itagaki H., Sakaguchi H., Miyazawa M., Ito Y., Suzuki H., Toyoda H. (2006). Development of an in vitro skin sensitization test using human cell lines: The human Cell Line Activation Test (h-CLAT): I. Optimization of the h-CLAT protocol. Toxicol. Vitr..

[121] Python F., Goebel C., Aeby P. (2007). Assessment of the U937 cell line for the detection of contact allergens. Toxicol. Appl. Pharmacol..

[122] Narita K., Ishii Y., Vo P.T.H., Nakagawa F., Ogata S., Yamashita K., Kojima H., Itagaki H. (2018). Improvement of human cell line activation test (h-CLAT) using short-time exposure methods for prevention of false-negative results. J. Toxicol. Sci..

[123] Edwards A., Roscoe L., Longmore C., Bailey F., Sim B., Treasure C. (2018). Adaptation of the human Cell Line Activation Test (h-CLAT) to animal-product-free conditions. ALTEX.

[124] Marigliani B., Silva J., Balottin L., Silva K., Baptista L., de Campos C.B.L., Augusto E.D.F.P. (2018). Adaptation of a skin sensitization assay to a chemically defined culture. Toxicol. Vitr..

[125] Dos Santos G.G., Reinders J., Ouwehand K., Rustemeyer T., Scheper R.J., Gibbs S. (2009). Progress on the development of human in vitro dendritic cell based assays for assessment of the sensitizing potential of a compound. Toxicol. Appl. Pharmacol..

[126] Ayehunie S., Snell M., Child M., Klausner M. (2009). A plasmacytoid dendritic cell (CD123+/CD11c−) based assay system to predict contact allergenicity of chemicals. Toxicology.

[127] Reuter H., Spieker J., Gerlach S., Engels U., Pape W., Kolbe L., Schmucker R., Wenck H., Diembeck W., Wittern K.-P. (2011). In Vitro detection of contact allergens: Development of an optimized protocol using human peripheral blood monocyte-derived dendritic cells. Toxicol. Vitr..

[128] Pépin E., Goutet M., Ban M. (2007). Murine bone marrow-derived dendritic cells as a potential in vitro model for predictive identification of chemical sensitizers. Toxicol. Lett..

[129] Battais F., Huppert C., Langonné I., Muller S., Sponne I. (2017). In Vitro detection of chemical allergens: An optimized assay using mouse bone marrow-derived dendritic cells. Contact Dermat..

[130] Takahashi T., Kimura Y., Saito R., Nakajima Y., Ohmiya Y., Yamasaki K., Aiba S. (2011). An In Vitro Test to Screen Skin Sensitizers Using a Stable THP-1–Derived IL-8 Reporter Cell Line, THP-G8. Toxicol. Sci..

[131] Nukada Y., Miyazawa M., Kosaka N., Ito Y., Sakaguchi H., Nishiyama N. (2008). Production of IL-8 in THP-1 cells following contact allergen stimulation via mitogen-activated protein kinase activation or tumor necrosis factor-alpha production. J. Toxicol. Sci..

[132] Parise C.B., Sá-Rocha V.M., Moraes J.Z. (2015). Skin sensitizer identification by IL-8 secretion and CD86 expression on THP-1 cells. Toxicol.Vitr..

[133] Toebak M.J., Pohlmann P.R., Sampat-Sardjoepersad S.C., von Blomberg B.M.E., Bruynzeel D.P., Scheper R.J., Rustemeyer T., Gibbs S. (2006). CXCL8 secretion by dendritic cells predicts contact allergens from irritants. Toxicol. Vitr..

[134] Johansson H., Lindstedt M., Albrekt A.-S., Borrebaeck C.A. (2011). A genomic biomarker signature can predict skin sensitizers using a cell-based in vitro alternative to animal tests. BMC Genom..

[135] Johansson H., Gradin R., Johansson A., Adriaens E., Edwards A., Zuckerstätter V., Jerre A., Burleson F., Gehrke H., Roggen E.L. (2019). Validation of the GARDTMskin Assay for Assessment of Chemical Skin Sensitizers: Ring Trial Results of Predictive Performance and Reproducibility. Toxicol. Sci..

[136] Zeller K.S., Forreryd A., Lindberg T., Gradin R., Chawade A., Lindstedt M. (2017). The GARD platform for potency assessment of skin sensitizing chemicals. ALTEX.

[137] Hooyberghs J., Schoeters E., Lambrechts N., Nelissen I., Witters H., Schoeters G., Heuvel R.V.D. (2008). A cell-based in vitro alternative to identify skin sensitizers by gene expression. Toxicol. Appl. Pharmacol..

[138] Lambrechts N., Vanheel H., Nelissen I., Witters H., Van Den Heuvel R., Van Tendeloo V., Schoeters G., Hooyberghs J. (2010). Assessment of Chemical Skin-Sensitizing Potency by an In Vitro Assay Based on Human Dendritic Cells. Toxicol. Sci..

[139] Neves B., Rosa S., Martins J., Silva A., Gonçalo M., Lopes M.C., Cruz M.T. (2013). Development of an in Vitro Dendritic Cell-Based Test for Skin Sensitizer Identification. Chem. Res. Toxicol..

[140] Nagahata T., Tsujino Y., Takayama E., Hikasa H., Satoh A. (2022). Evaluation of skin sensitization based on interleukin-2 promoter activation in Jurkat cells. Biomed. Rep..

[141] Richter A., Schmucker S.S., Esser P.R., Traska V., Weber V., Dietz L., Thierse H.-J., Pennino D., Cavani A., Martin S.F. (2013). Human T cell priming assay (hTCPA) for the identification of contact allergens based on naive T cells and DC-IFN-γ and TNF-α readout. Toxicol. Vitr..

[142] Vocanson M., Achachi A., Mutez V., Tailhardat M., Le Varlet B., Rozières A., Fournier P., Nicolas J.-F. (2014). Human T Cell Priming Assay: Depletion of Peripheral Blood Lymphocytes in CD25+ Cells Improves the In Vitro Detection of Weak Allergen-Specific T Cells. EXS.

[143] Claesson M.H., Dissing S., Tscherning T., Geisler C. (1993). T-cell activation. V. Anti-major histocompatibility complex class I antibody-induced activation and clonal abortion in Jurkat T-leukaemic cells. Immunology.

[144] Hou F., Xing C., Li B., Cheng J., Chen W. (2020). Performance of a novel In Vitro assay for skin sensitization based on activation of T lymphocytes. ALTEX.

[145] Thélu A., Catoire S., Kerdine-Römer S. (2020). Immune-competent in vitro co-culture models as an approach for skin sensitisation assessment. Toxicol. Vitr..

[146] Balszuweit F., Menacher G., Bloemeke B., Schmidt A., Worek F., Thiermann H., Steinritz D. (2014). Development of a co-culture of keratinocytes and immune cells for in vitro investigation of cutaneous sulfur mustard toxicity. Chem. Biol. Interact..

[147] Hennen J., Blömeke B. (2017). Keratinocytes improve prediction of sensitization potential and potency of chemicals with THP-1 cells. ALTEX.

[148] Frombach J., Sonnenburg A., Krapohl B.-D., Zuberbier T., Peiser M., Stahlmann R., Schreiner M. (2018). Lymphocyte surface markers and cytokines are suitable for detection and potency assessment of skin-sensitizing chemicals in an in vitro model of allergic contact dermatitis: The LCSA-ly. Arch. Toxicol..

[149] Lee S., Greenstein T., Shi L., Maguire T., Schloss R., Yarmush M. (2018). Tri-culture system for pro-hapten sensitizer identification and potency classification. Technology (Singap. World Sci.).

[150] Karri V., Lidén C., Fyhrquist N., Högberg J., Karlsson H.L. (2021). Impact of mono-culture vs. Co-culture of keratinocytes and monocytes on cytokine responses induced by important skin sensitizers. J. Immunotoxicol..

[151] Eskes C., Hennen J., Schellenberger M.T., Hoffmann S., Frey S., Goldinger-Oggier D., Peter N., van Vliet E., Blömeke B. (2019). The HaCaT/THP-1 Cocultured Activation Test (COCAT) for skin sensitization: A study of intra-lab reproducibility and predictivity. ALTEX.

[152] Galbiati V., Maddalon A., Iulini M., Marinovich M., Corsini E. (2020). Human keratinocytes and monocytes co-culture cell system: An important contribution for the study of moderate and weak sensitizers. Toxicol. Vitr..

[153] Meloni M., De Servi B., Le Varlet B. (2010). New approach for chemical sensitizing potential assessment using THP-1 and NCTC 2544 co-culture. ALTEX.

[154] Hennen J., Aeby P., Goebel C., Schettgen T., Oberli A., Kalmes M., Blömeke B. (2011). Cross talk between keratinocytes and dendritic cells: Impact on the prediction of sensitization. Toxicol. Sci..

[155] Cao Y.P., Ma P.C., Liu W.-D., Zhou W.-Q., Tao Y., Zhang M.-L., Li L.-J., Chen Z.-Y. (2012). Evaluation of the skin sensitization potential of chemicals in THP-1/keratinocyte co-cultures. Immunopharmacol. Immunotoxicol..

[156] Sawada Y., Tsukumo H., Fukuda J., Iijima K., Itagaki H. (2022). Co-Culture of THP-1 Cells and Normal Human Epidermal Keratinocytes (NHEK) for Modified Human Cell Line Activation Test (h-CLAT). Appl. Sci..

[157] Chanput W., Mes J.J., Wichers H.J. (2014). THP-1 cell line: An in vitro cell model for immune modulation approach. Int. Immunopharmacol..

[158] Kaur G., Dufour J.M. (2012). Cell lines: Valuable tools or useless artifacts. Spermatogenesis.

[159] Schreiner M., Peiser M., Briechle D., Stahlmann R., Zuberbier T., Wanner R. (2007). A loose-fit coculture of activated keratinocytes and dendritic cell-related cells for prediction of sensitizing potential. Allergy.

[160] Koppes S.A., Engebretsen K.A., Agner T., Angelova-Fischer I., Berents T., Brandner J., Brans R., Clausen M.-L., Hummler E., Jakasa I. (2017). Current knowledge on biomarkers for contact sensitization and allergic contact dermatitis. Contact Dermat..

[161] Ohtani T., Mizuashi M., Nakagawa S., Sasaki Y., Fujimura T., Okuyama R., Aiba S. (2009). TGF-beta1 dampens the susceptibility of dendritic cells to environmental stimulation, leading to the requirement for danger signals for activation. Immunology.

[162] Miyazawa M., Ito Y., Yoshida Y., Sakaguchi H., Suzuki H. (2007). Phenotypic alterations and cytokine production in THP-1 cells in response to allergens. Toxicol In Vitro.

[163] Sakaguchi H., Ashikaga T., Miyazawa M., Yoshida Y., Ito Y., Yoneyama K., Hirota M., Itagaki H., Toyoda H., Suzuki H. (2006). Development of an in Vitro skin sensitization test using human cell lines; human Cell Line Activation Test (h-CLAT). II. An inter-laboratory study of the h-CLAT. Toxicol. Vitr..

[164] Schellenberger M.T., Bock U., Hennen J., Groeber-Becker F., Walles H., Blömeke B. (2019). A coculture system composed of THP-1 cells and 3D reconstructed human epidermis to assess activation of dendritic cells by sensitizing chemicals after topical exposure. Toxicol. Vitr..

[165] Bock S., Said A., Müller G., Schäfer-Korting M., Zoschke C., Weindl G. (2018). Characterization of reconstructed human skin containing Langerhans cells to monitor molecular events in skin sensitization. Toxicol. Vitr..

[166] Russell W.M.S., Burch R.L. (1959). The Principles of Humane Experimental Technique.

[167] OECD (2017). Guidance Document on the Reporting of Defined Approaches and Individual Information Sources to Be Used within Integrated Approaches to Testing and Assessment (IATA) for Skin Sensitisation [Internet]. https://www.oecd-ilibrary.org/content/publication/9789264279285-en.

